# Addenbrooke's Hospital, Cambridge

**Published:** 1917-05-26

**Authors:** 


### Addenbrooke's Hospital, Cambridge.

PROCEEDINGS OF QUARTERLY COURT.

At the Quarterly Court held on May 14 it was reported that the expenditure for the first quarter of the year was £4,399, as compared with £3,542 in 1916. As there had been an increase of sixty-seven in the daily average number of beds occupied on the civil and military sides together, the rise in the cost of maintenance was not unexpected.

There was a slight diminution in the number of out-patients, but an increase in casualty and dental patients.

During Easter-time an appeal was made to schools for collections of new-laid eggs for the use of the patients, which met with a generous response. At some schools these collections are made weekly. To the regret of the committee, during this quarter Mr. G. Wherry, M.A., M.C. (Cantab.), F.R.C.S. England, who had been attached to the hospital for many years, resigned the post of senior honorary surgeon. Mr. W. H. Bowen, M.B., M.S. London, F.R.C.S. England, has been appointed honorary assistant surgeon in the out-patient department, and in charge of the ear and throat department.

The term of office of Dr. J. B. Bradbury, senior honorary physician, having expired, his offer to continue his services during the war has been gratefully accepted.

Major Arthur Cooke, honorary surgeon, having been mobilised for military service abroad, Mr. Geo. Wherry, honorary consulting surgeon, has kindly arranged to help the hospital in this emergency.

The governors sanctioned the admission of patients, for whom a charge will be made for their treatment, under the proposed scheme for dealing with cases of venereal disease, and authorised the general committee to complete the requisite arrangements for the establishment of a venereal disease treatment centre at the hospital.

---

Addenbrooke's Hospital, Cambridge, has had a valuable crop of legacies recently. Mrs. Barnett has by her will bequeathed to the hospital the residue of her estate, which according to valuation for probate amounts to £8,100, though the ultimate figure is uncertain. The deceased was in her life a generous benefactor to the hospital, and endowed its pathological laboratory and *post-mortem* department in memory of her son, who was for many years secretary to the institution. Dr. T. B. Brookes-Bumpsted, of Trumpington, another most generous benefactor, has by his will bequeathed the sum of £2,000, duty free, to the endowment fund; and Mr. Joseph Turner Bailey, deceased, has by his will, subject to the life interest of two friends, bequeathed his estate, consisting of property bringing in from £13 to £15 per week, to Addenbrooke's Hospital.

